# Development and Reliability of Brief Dietary Assessment Tools for Hispanics

**Published:** 2006-06-15

**Authors:** Patricia Wakimoto, Gladys Block, Shelly Mandel, Norma Medina

**Affiliations:** National Center for Minority Health and Health Disparities, Children’s Hospital Oakland Research Institute; University of California, Berkeley, School of Public Health, Division of Community Health and Human Development, Berkeley, Calif; University of California, Berkeley, School of Public Health, Division of Community Health and Human Development, Berkeley, Calif; University of California, Berkeley, School of Public Health, Division of Community Health and Human Development, Berkeley, Calif

## Abstract

**Introduction:**

The Hispanic population is the most rapidly growing ethnic group in the United States. Culturally appropriate and efficient strategies for dietary assessment for this population are currently lacking. To address this issue and promote a healthy diet for disease prevention, we developed screening tools to assess the fruit, vegetable, and fat intake of Mexican Americans.

**Methods:**

Brief screening tools (screeners) were developed based on national data on Mexican Americans' dietary intake and were then modified after interviews and field testing. The screeners take less than 10 minutes to administer. A reliability study was conducted from June through September 2000, during which 93 Mexican Americans (39 men, 54 women) completed the screeners twice, 1 month apart. The mean age of the study participants was 36.5 years (range 18–71 years), and 91.4% had been born in Mexico.

**Results:**

Correlations between the first and second administration of the screeners were r = 0.64 for fruits and vegetables and r = 0.85 for dietary fat contributors. In addition, estimates of fruit and vegetable consumption frequency were similar to statewide estimates for Hispanics in California. Reproducibility of reported use of vitamin supplements at least once per week was high; 84% were classified in the same way both times (*P* < .001).

**Conclusion:**

The screening tools provide a reliable assessment of selected dietary factors among Mexican Americans. The tools can be scored immediately to provide feedback to respondents. They may be useful in situations requiring easily administered and economical assessment tools, such as in large-scale studies or in community situations.

## Introduction

Any effort to improve public health in the United States must consider the diversity of the U.S. population and the varying disease incidence among ethnic groups. Among the ethnic groups in the United States, the Hispanic population is the most rapidly growing. Hispanics constitute 32.4% of the population in California and 13.0% of the U.S. population overall. Currently, 37.4 million Hispanics are living in the United States — one in eight people (1). By the year 2050, it is estimated that the Hispanic population will increase to 102 million, or 24.5% of the U.S. population (2), one of the major changes that is occurring in the composition of the U.S. population. The population shift has been accompanied by substantial health disparities. In terms of age-adjusted years of potential life lost, Hispanics experience disproportionately more strokes, cervical cancer, perinatal conditions, and other conditions (2). Conditions related to dietary habits have taken a particularly heavy toll. Age-adjusted years of potential life lost due to diabetes is 41% greater among Hispanics than among non-Hispanic whites; the rates of overweight and obesity are 11% and 7% higher among Hispanic men and 26% and 32% higher among Hispanic women than among non-Hispanic whites. Thus, accurate and easy ways to assess the dietary habits of the rapidly growing Hispanic communities in the United States are needed, as are appropriate strategies that promote healthy lifestyles and prevent disease.

Although numerous studies have investigated the relationship between dietary intake and disease prevention, many of them were conducted with the non-Hispanic population, so it is unclear whether the conclusions are generalizable to other ethnic groups. Many of the studies relied on dietary assessment instruments that are widely used and accepted in the non-Hispanic population. However, appropriate dietary assessment tools suitable for studying ethnic minority communities in the United States are lacking. Developing and validating such research-appropriate tools are key steps toward addressing health issues of the Hispanic population. In addition, because of the limited education level of many Hispanics, brief screening tools are needed that can be administered in a few minutes or self-administered in community settings.

We developed brief fat and fruit and vegetable intake screening tools (screeners) appropriate for a subpopulation of the Hispanic community and conducted a reliability study of the screeners in a population of Mexican Americans in California. In addition, we used the instruments in community settings and for health education. The screeners are easy to administer or can be self-administered and take only a few minutes to complete. The scoring system is simple, and the participant receives immediate feedback on dietary intake of fat and fruit and vegetables.

## Methods

The food list for the two screeners was developed using previously described methods (3). The list was created based on national data on the Mexican American population from the Third National Health and Nutrition Examination Survey (NHANES III) (4), which included an oversampling of the Mexican American population. For the fruit and vegetable screener, we included the foods most commonly eaten. For the fat screener, we included the foods making the largest contribution to grams-of-fat intake in this population, including foods contributing the top 60% of all fat intake, which has been the standard approach in developing Block screeners (3). This approach has produced good correlations with longer food lists and with reference data. We also asked a simple question about vitamin supplement usage during the previous week.

After creating a food list based on NHANES III, we conducted additional development and testing in three phases: interviews and focus groups, field testing, and a reliability study. For all three phases, participants were recruited from community-based organizations primarily serving the low-income Hispanic population, including a community center, local health clinics, and adult education classes (including English as a Second Language classes). The study was conducted from June through September 2000.

During the first phase, individual interviews and small group discussions were conducted to obtain information on cultural values, beliefs, and behaviors related to food, nutrition, and health and to refine the food list and instructions. Thirty-five individual interviews were conducted, and a total of 70 men and women participated in the small group discussions. Discussions were conducted in Spanish or English, depending on the preference of the participants. Participants were from both rural and urban areas and were primarily Mexican and Mexican Americans. Participants completed the two screeners, and we obtained feedback about the screeners' ease of administration (when self-administered), format, appropriateness of food wording, and usefulness as dietary assessment instruments.

Field tests were conducted as a community service and were used as an opportunity to informally observe the screeners' usability in real-world situations. Several hundred people participated at health fairs and other community gatherings. The screeners were administered and scored immediately, and feedback about participants' fat and fruit and vegetable intakes was provided. Administration of the screener took 5 to 10 minutes. Participants were asked for feedback about their satisfaction with the screener and its results as well as its ease of use.

For the reliability study, participants were recruited from three community-based organizations, including an organization providing referral services, adult education classes, and two health clinics (one urban and one semirural). Interviewers administered the two screeners (fat intake and fruit and vegetable intake) twice, 1 month apart.

We examined results using two different scoring systems, *simple* and *times per week*. The simple method scored the frequency responses as 0 to 4 across the five categories of the fat screener or 0 to 5 across the six categories of the fruit and vegetable screener. Thus, for the simple fat score, each food was given a score from 0 to 4, and then the scores were summed for all the 16 fat screener foods. The same procedure was followed for the fruit and vegetable screener. The advantage of the simple (0 to 4) scoring system is that it only involves adding one-digit numbers and is therefore easier to self-score. The times-per-week scoring system used the reported frequency of each category to assign the score. For example, 2 to 3 times per week was assigned a score of 2.5. The times-per-week scores for each food were summed using all foods in the fat or fruit and vegetable screeners, resulting in an estimate of times per week for each screener. This scoring system is not appropriate for self-scoring but does permit an estimate of servings per week.

Descriptive statistical analyses were performed. For the reliability analysis, agreement between the two administrations was assessed using Pearson correlation coefficients. Both scores were approximately normally distributed and were not transformed. To estimate the meaning of the simple score in relation to recommended levels of intake, we also conducted a regression analysis of the relationship between the simple score and estimated times per day.

The University of California Committee for the Protection of Human Subjects approved the research, and participants signed informed consent forms. 

## Results

### Interviews and field testing

Based on the individual interviews and small group discussions, only minor modifications were made to the original food list. The original food list derived from the national data was found to represent the foods important to the study population. Formatting changes were made and words were added or modified to clarify certain food items. Overall, the participants reported that the screeners were easy to complete and that the process helped them evaluate their diets.

The fat screener had 16 items, including eggs, whole milk, flour tortillas, hamburgers, tacos and burritos, other mixed dishes with meat, pork, fried chicken, cheese, pizza, refried beans, French fries, chips, cake, fat in cooking, and salad dressing. The fat screener had five frequency response categories, from once a month or less to five or more times per week. The fruit and vegetable screener had 7 items (Appendices), including fruit juice, other fruit, green salad, tomatoes or salsa, potatoes, soups or stews with vegetables, and any other vegetables. The fruit and vegetable screener had six frequency response categories, from less than once per week to two or more times per day. The frequency categories are the same as those used in the English versions of Block screening questionnaires (3,5). Portion sizes were not assessed.

For the interviews and field testing of the screeners, almost 300 participants were included, of whom 49% were men. The majority of the respondents were married. Age distribution was as follows: 38% were younger than 30 years of age, 47% were between 30 and 49 years of age, and 15% were 50 years or older. Eighty-five percent of the participants completed the Spanish-language version, and 15% chose to complete the English version. Almost 42% had an eighth-grade education or less, and 21% had completed high school (data not shown).

### Reliability study

For the reliability study, 93 people completed the two brief questionnaires twice, 4 weeks apart. Men comprised 42% of the sample, and 91% of participants had been born in Mexico (Table 1).

To estimate the average frequency of consumption, we used the times-per-week scoring system. The average consumption frequency of fruits and vegetables was 4.2 times per day at the first visit (Table 2). On the first administration of the screener, 32% of participants scored at 5 or more times per day, and 30% scored at fewer than 3 times per day (data not shown). On average, fat sources were consumed 3.8 times per day. Twenty-four percent of participants reported eating fat sources 5 or more times per day, and 14% reported eating them fewer than twice a day (data not shown). Men and women both reported a lower intake of fruits, vegetables, and fat on the second administration.

During the first administration of the screener, 44% of participants reported consuming vitamin supplements at least once per week. Women reported similar usage during the first and second screening. In contrast, 42% of men in the first screening reported consuming vitamins at least once per week, whereas only 30% of men participating in the second screening reported consuming vitamins at least once per week. However, this difference between men and women may not be meaningful because of the small sample size; only 5 of the 39 men gave a different answer on the first and second screenings.

The reliability correlations showed no important differences between using the simple and times-per-week scoring systems, so results for the reliability analysis are based on the simple scoring system. The simple scoring system was a continuous variable ranging from 1 to 28 in this sample. The reliability correlation was r = 0.64 for the fruit and vegetable screener and r = 0.85 for the fat screener (Table 3). The slightly lower correlations for fruits and vegetables could have been caused by variations in their availability because the data collection spanned the months from June through September. Adjustments for age and sex had little effect on the correlation coefficients. The screeners also asked about use of vitamin supplements "at least once a week." Reproducibility of this question was also high; 84% were classified in the same way both times (*P* < .001) (Table 3).

The two scoring systems were highly correlated (r = 0.99 for the fat scores in the first administration). Regression of the first-administration times-per-day score on the simple score yielded the following equations:

Fat Times/Day = 0.16 × Fatsimple - 0.48

FV Times/Day = 0.34 × FVsimple - 1.34,

where FV indicates fruit and vegetable; Fatsimple, *simple* fat score; and FVsimple, *simple* fruit and vegetable score. Using these equations, we created usable cutoff points from the simple scoring systems. For fruits and vegetables, the simple scores were broken down as follows:

- ≥18 ≈ 5 per day, Excellent
- 16–17 ≈ 4 per day, Good
- 13–15 ≈ 3 per day, Fair
- <13 ≈ per day, Poor

In the first administration of the first screener, 46% had fruit and vegetable scores of excellent; 12%, good; 14%, fair; and 28%, poor. In the second administration, scores were 28%, excellent; 15%, good; 20%, fair; and 28%, poor — a more likely distribution.

For fats, the cutoff points were more arbitrary. We used simple quartiles based on the distribution from the second administration. This approach may be better than estimating percentage of energy from fat, because a low fat percentage may simply be the result of an overall high energy intake or a high energy intake from soft drinks or alcohol. Following is the score breakdown for the fat screener:

- ≤18 = Excellent
- 19–24 = Good
- 25–33 = Fair
- >33 = Poor

## Discussion

Our analyses have shown that the two brief dietary screeners for Mexican Americans have good reliability. We also tested the screeners in community situations such as health fairs, community centers, and clinics and found them to be well-received and to provide useful information for the participants.

Many brief dietary assessment tools have been developed over the past few decades, most of which have been simplified food frequency questionnaires (FFQs) or questionnaires focusing on eating behaviors. Several were developed to assess dietary fat intake or percentage of energy from fat (3,6-8). A review of validation studies of fat screeners was conducted by Yaroch et al (9). Our correlation of r = 0.85 (Table 3) is comparable to the correlation of r = 0.87 found among white middle-class participants for the Kristal et al Food Habits Questionnaire (10) and the Connor et al Diet Habit Survey (11).

Brief tools assessing fruit and vegetable intake have also been developed, evaluated, and reviewed (12-15). Our results of r = 0.64 for the fruit and vegetable screener and r = 0.85 for the fat sources screener are similar to the r = 0.62 for a composite index of diet atherogenicity based on the 17 food items, found by Shea et al for the 17-item dietary component of the Behavioral Risk Factor Surveillance System conducted among white, African American, and Hispanic respondents (16). Smith-Warner et al (17) reported reliability correlations of r = 0.55 and higher.

Few brief screeners have been used with substantial numbers of Mexican Americans, and none (of which we are aware) were systematically developed for this population based on national data. The few Spanish-language screeners and brief tools that have been used with large numbers of Hispanic adults include 1) the National Cancer Institute's 5 A Day for Better Health fruit and vegetable screener (18) used in the Centers for Disease Control and Prevention state and local Behavioral Risk Factor Surveillance System (BRFSS) (19) and the California Health Interview Survey (CHIS) (20); 2) a modification of the Food Habits Questionnaire originally developed by Kristal et al (10)); and 3) the fruit, vegetable, and fat questions of the Food Behavior Checklist (21-24).

Some authors have investigated the reliability of longer instruments in assessing the dietary intake of Hispanics in the United States. Taren et al (25) studied the reliability of the 159-item Southwest FFQ, a modification of the Block FFQ, among 79 Mexican American and 80 non-Hispanic participants. The second FFQ was administered after 2 weeks. The average reliability coefficient for energy and fats was r = 0.81, similar to our r= 0.85 for the fat screener. The average reliability coefficient for vitamin C, folate, beta-carotene, and lycopene was r = 0.72, similar to our fruit and vegetable screener correlation of r = 0.64. Mayer-Davis et al studied the reliability of the Insulin Resistance Atherosclerosis Study (IRAS) FFQ (also a modification of the Block questionnaire) among 43 rural Hispanic women after a 2- to 4-year interval (26). Reliability coefficients for energy and fats ranged from r = 0.54 to r = 0.64, and for vitamin C the correlation was r = 0.59. Cullen et al studied the reliability of the Youth/Adolescent Questionnaire among 41 Hispanic seventh- and eighth-grade students after a 3-week interval (27). Questions were read to the students in class. The reliability coefficient for energy was r = 0.61 and for servings of fruits, vegetables, and juices was r = 0.68.

For some research purposes, full-length dietary questionnaires are preferable because they provide estimates for a range of nutrients. However, some situations call for self-administration, but among populations with lower education levels (such as the Mexican American sample in this study), self-administration of long questionnaires is problematic. In a multiethnic validation study of self-administered full-length questionnaires among low-income participants in the Special Supplemental Nutrition Program for Women, Infants, and Children (WIC) (28), researchers found that validity was acceptable among whites and African Americans but not among Hispanics. In addition, in some research situations, such as interventions to increase fruit and vegetable intake or decrease fat intake, a brief, targeted instrument may be all that is needed. 

For interventions designed to improve dietary habits, it is necessary to know the sensitivity of the instrument in detecting change. The sensitivity can be calculated from the data presented here. The following formula estimates the sample size needed to detect change within a single group:

N = ([Z_α/2_ + Z_β_]2^2^ × σ^2^_d_) / Δ^2^ 

where Z_α/2_ indicates the upper α/2 percent point of the normal distribution; Z_β_, the lower β percent point of the normal distribution; σ^2^_d_, the variance of the differences in the *before* and *after* estimates, which in this analysis was 2.407 for fruits and vegetables per day and 0.959 for fat sources per day; and Δ, the size of the change you would like to achieve.

For example, to detect a change of 0.5 times per day in fat sources and fruit and vegetables sources, with a two-sided alpha and 90% power, would require sample sizes of 41 (for fat) and 102 (for fruit and vegetables). The sample sizes needed to detect the difference between the *changes* in two groups (e.g., intervention and control) can be calculated as follows:

N_each_ = ([Z_α/2_ + Z_β_]^2^ × 2 × σ^2^_d_) / Δ_d_^2^ ,

where Z_α/2_ indicates the upper α/2 percent point of the normal distribution; Z_β_, the lower β percent point of the normal distribution; σ^2^_d_, the variance of the differences in the *before* and *after* estimates; Δ_d_, the difference between the *two change scores*.

For example, if the control group increases by 0.25 times per day and the intervention group increases by 0.75 times per day, the Δ_d_ is 0.50. The study would need 81 and 203 people in each group to detect that degree of difference in the changes in the two groups for fat intake and fruit and vegetable intake, respectively.

The reason for the lower intake estimates for fat sources and fruits and vegetables in the second administration of the screeners is unclear. However, this pattern has been observed repeatedly (although not exclusively) in dietary data, with both FFQs and 24-hour recalls (27). Given this pattern of respondent behavior, it would be prudent to administer a baseline questionnaire twice and use the second administration as the estimate of *before* in an intervention study.

Our data comparing fruit, vegetable, and fat intakes and supplement use in two questionnaire administrations to 93 individuals demonstrate good reliability. The fact that the sample included both men and women and comprised a population whose primary language was Spanish suggests broad usefulness among U.S. Hispanic Americans, of whom 66.9% are of Mexican origin (29). In addition, most of the food names used in the screeners are commonly used among all U.S. Hispanic populations. However, the cultural diversity in the U.S. Hispanic population suggests the desirability of testing the instrument among Cuban, Puerto Rican, and Central American subgroups. 

The suggested cutoff points for the simple scoring system may be used to give immediate feedback to respondents when the screeners are scored by the respondents themselves or by an assistant. The resulting estimated fruits and vegetables times per day from the time-per-week system and the corresponding simple system may be interpreted to approximately equal servings per day, because one piece of fruit equals one serving, and most vegetables are not eaten more than once per day. The times-per-day estimates of 4.2 at the first administration and 3.6 at the second administration correspond reasonably well with estimates of 3.8 servings of fruits and vegetables per day among Hispanic adults in a 1999 study in California (30).

For the fat-intake screener, either scoring system translates to times per day, and the foods often have no recommended serving size or frequency. Furthermore, the fat-intake scores are based on fat grams, whereas recommendations are based on percent of calories. However, during the development of the original English-language screener for fat intake, it was shown that the screener score based on fat grams correlated at r = 0.54 with percentage of calories from fat as estimated by the mean of three 4-day records (3). Thus, the fat-intake score (either simple or times per week) may be used as a continuous variable to rank participants' fat intake.

In addition to reliability, another key feature of the brief screeners is ease of administration. Although screeners are not substitutes for more comprehensive dietary assessment instruments that assess the entire diet, they fill the practical need for easily administered, economical, and less burdensome tools. These features are particularly relevant to the practical use of these tools among larger populations and in community settings. Our preliminary results with community-based populations show the screeners to be well-received. Screeners can identify individuals at high risk of disease due to too-frequent consumption of high-fat foods or infrequent consumption of fruits and vegetables. Because they are simple, easy to administer, and easy to understand, screeners can be useful in community settings where precise or accurate estimates are unnecessary. The screeners themselves can provide content for nutrition education programs. With their immediate feedback on dietary intake, the screeners can stimulate respondents' interest, which may facilitate dietary change. When resources are limited, or among subpopulations such as minority populations or those with limited English language proficiency, brief screeners can be useful instruments for dietary assessment, raise awareness of individuals' food intakes, and heighten interest in and motivation for making changes.

## Figures and Tables

**Table 1:** Participant Characteristics in Study to Determine Reliability of a Dietary Screener for Mexican Americans (N = 93), Berkeley, Calif, 2000

**Characteristic**	**Value**
**Sex, No. (%)**	
Male	39 (42)
Female	54 (58)
**Birthplace, No. %**	
Mexico	85 (91.4)
South America	2 (2.2)
United States	6 (6.5)
**Age, y, mean (SD)**	36.5 (14.5)

**Table 2:** Mean Fruit and Vegetable, Fat, and Supplement Consumption, by Screening Administration (N = 93) Berkeley, Calif, 2000

**Food or Supplement**	**First Screening**	**Second Screening**
**Fruits and vegetables (7 food items), mean times/day (SD)**		
All	4.2 (1.7)	3.6 (1.7)
Men	3.9 (1.8)	3.3 (1.6)
Women	4.4 (1.6)	3.9 (1.8)
**Fat contributors (16 food items), mean times/day (SD)**		
All	3.8 (1.7)	3.5 (1.7)
Men	4.2 (1.7)	3.9 (1.6)
Women	3.5 (1.7)	3.3 (1.7)
**Vitamin supplements ≥ 1 time/week, %**		
All	44	40
Men	42	30
Women	45	46

**Table 3:** Screener Reliability Correlations and Vitamin Supplement Agreement (N = 93), Berkeley, Calif, 2000

**Food or Supplement**	**Correlation or Agreement^a^**	***P *Value**
Fruit and vegetable score correlation	r = 0.64	<.001
Fat score correlation	r = 0.85	<.001
Vitamin supplements ≥1 time/week, % agreement	84	<.001

a Correlation between scores from the first and second screener administration was determined using the Pearson product moment correlation statistic.

## Appendices

These screeners may be reproduced and used freely. Computerized scanning and scoring and online administration are available from: www.nutritionquest.com.

### Appendix A: English Screeners

Think about your eating habits over the past month or so. About how often do you eat each of the following foods either at home or in restaurants? Mark an "x" in **one** box for each food.

**Table:**

**How often do you eat. . .**	**[A] Once per MONTH or less**	**[B] 2-3 times per MONTH**	**[C] 1-2 times per WEEK**	**[D] 3-4 times per WEEK**	**[E] 5 or more times per WEEK**
Eggs	□	□	□	□	□
Whole milk or chocolate milk(not low fat or skimmed)	□	□	□	□	□
Flour tortillas (not corn)	□	□	□	□	□
Hamburgers or cheeseburgers	□	□	□	□	□
Tacos, burritos, or enchiladas	□	□	□	□	□
Other mixed dishes with meat	□	□	□	□	□
Roast pork or chops, roast beef, or steak	□	□	□	□	□
Fried chicken	□	□	□	□	□
Cheese or cheese spreads	□	□	□	□	□
Pizza	□	□	□	□	□
Refried beans	□	□	□	□	□
French fries or fried potatoes	□	□	□	□	□
Potato chips, corn chips, or peanuts	□	□	□	□	□
Cake, sweet rolls, doughnuts, or Mexican sweet bread	□	□	□	□	□
How often do you use fat or oil to fry, cook, or season?	□	□	□	□	□
Salad dressing	□	□	□	□	□

**Table:**

		To score:		Total fat score =	
How many As?		× 0 =			0-18 Very good! Congratulations. 19-24 Quite good! 25-33 Not so good. 34+ Could do better! Eat less fat and more fruits and vegetables.
How many Bs?		× 1 =			
How many Cs?		× 2 =			
How many Ds?		× 3 =			
How many Es?		× 4 =			
	Total	=			

Think about your eating habits over the past month or so. About how often do you eat each of the following foods either at home or in restaurants? Mark an "x" in **one** box for each food.

**Table:**

**How often do you eat…**	**[A] Less than once per WEEK**	**[B] About 1 time per WEEK**	**[C] 2-3 times per WEEK**	**[D] 4-6 times per WEEK**	**[E] Once per DAY**	**[F] 2 or more times per DAY**
Fruit juice, like orange, apple, grape, fresh, frozen or canned (not soda or other drinks)	□	□	□	□	□	□
Not counting juice, how often do you eat any fruit — fresh, cannned, or in smoothies?	□	□	□	□	□	□
Green salad (like lettuce or spinach salad)	□	□	□	□	□	□
Tomatoes or salsa fresca	□	□	□	□	□	□
Vegetable soup or stew with vegetables	□	□	□	□	□	□
Potatoes, any kind, including baked, mashed, or French fried	□	□	□	□	□	□
Any other vegetables, including green beans, peas, corn, broccoli, or any other	□	□	□	□	□	□

Do you take vitamin or mineral supplements at least once a week?

□ Yes     □ No

Age: _____     Sex: □ Male     □ Female

Where were you born?

□ Mexico     □ Central America     □ South America     □ United States     □ Other place

**Table:**

		To score:		
How many As?		× 0 =		
How many Bs?		× 1 =		
How many Cs?		× 2.5 =		
How many Ds?		× 5 =		
How many Es?		× 7 =		
How many Fs?		× 14 =		
	Total	= ÷ 7 =		

Estimated portions of fruits and vegetables per day =  _________

**Note:** If the fat screener and fruit and vegetable screener are used separately, obtain demographic and supplement intake information on each.

### Appendix B: Spanish Screeners

Piense en sus hábitos de alimentación en el último mes. Con qué frecuencia ha comido los siguientes alimentos?

Marque la frecuencia con **una** “X” en el cuadro para cada alimento. Incluya alimentos que comió en casa o en restaurantes.

**Table:**

**Con qué frecuencia come usted…**	**[A] Una vez por MES o menos**	**[B] 2-3 veces por MES**	**[C] 1-2 veces por SEMANA**	**[D] 3-4 veces por SEMANA**	**[E] 5 o mas veces por SEMANA**
Huevos	□	□	□	□	□
Leche entera o leche con chocolate (No leche semi-descremada-1%-2% o descremada)	□	□	□	□	□
Tortillas de harina (no de maíz)	□	□	□	□	□
Hamburguesas o hamburguesas con queso	□	□	□	□	□
Tacos, burritos o enchiladas	□	□	□	□	□
Otros alimentos mezclados con carne	□	□	□	□	□
Puerco/cerdo, asado o chuletas, o res asado, o bistec	□	□	□	□	□
Pollo frito	□	□	□	□	□
Queso o queso para untar	□	□	□	□	□
Pizza	□	□	□	□	□
Frijoles refritos	□	□	□	□	□
Papas a la francesa o papas fritas	□	□	□	□	□
Papitas, chips de maíz, o cacahuates	□	□	□	□	□
Pastel, roles de dulce, donas, o pan dulce (Mexicano)	□	□	□	□	□
Con que frecuencia usa grasa o aceite para freir, cocer o sazonar sus alimentos	□	□	□	□	□
Aderezos o salsa para ensaladas	□	□	□	□	□

**Table:**

		Para Contar:		Grasa Total =	
¿Cuántas As?		× 0 =			0-18 Es un campeón! Adelante.
¿Cuántas Bs?		× 1 =			19-24 Bastante bien.
¿Cuántas Cs?		× 2 =			25-33 No muy bueno.
¿Cuántas Ds?		× 3 =			34+ Un poco malo. Reduzca la grasa y coma más frutas y vegetales.
¿Cuántas Es?		× 4 =			
	Total	=			

Piense en sus hábitos de alimentación en el último mes. Con qué frecuencia ha comido los siguientes alimentos?

Marque la frecuencia con **una** "X" en el cuadro para cada alimento.

**Table:**

**Con qué frecuencia come usted…**	**[A]Menos de una vez por SEMANA**	**[B]1 vez por SEMANA**	**[C]2-3 veces por SEMANA**	**[D]4-6 veces por SEMANA**	**[E]1 vez por DÍA**	**[F]2 o mas veces por DÍA**
Jugo de fruta, como de naranja, manzana, o uva — naturales, congelados, o de lata o en aguas frescas (**no** otros refrescos u bebidas gaseosas)	□	□	□	□	□	□
Sin contar jugos, con que frecuencia come frutas — naturales, o de lata, congelada o en licuados	□	□	□	□	□	□
Ensalada verde (como de lechuga o espinacas)	□	□	□	□	□	□
Tomates o salsa fresca	□	□	□	□	□	□
Sopas de verduras o caldos con verduras	□	□	□	□	□	□
Papas, de cualquier tipo incluyendo horneadas, puré o a la francesa	□	□	□	□	□	□
Algunas otra verduras, incluyendo ejotes o habichuelas verdes, repollo, elote o mazorca (maíz), o brócoli algun otro	□	□	□	□	□	□

Está tomando vitaminas o suplementos minerales por lo menos una vez a la semana?

□ Si     □ No

Edad: _____     Sexo: □ Hombre     □ Mujer

Es de:

□ Mexico     □ Centro America     □ Sudamerica     □ los Estados Unidos     □ o de otra lugar

**Table:**

		Para Contar:		
¿Cuántas As?		× 0 =		
¿Cuántas Bs?		× 1 =		
¿Cuántas Cs?		× 2.5 =		
¿Cuántas Ds?		× 5 =		
¿Cuántas Es?		× 7 =		
¿Cuántas Fs?		× 14 =		
	Total	= ÷ 7 =		

Porciones aproximadas de **frutas y vegetales** al día=  _________

**Note:** If the fat screener and fruit and vegetable screener are used separately, obtain demographic and supplement intake information on each.
